# Diuretic resistance and the efficacy of hydrochlorothiazide in acute decompensated heart failure: A post‐hoc analysis of the CLOROTIC trial

**DOI:** 10.1002/ejhf.70002

**Published:** 2025-08-26

**Authors:** Ambarish Pandey, Matthew W. Segar, Neil Keshvani, Pau Llácer, Jesús Casado, José Luis Morales‐Rull, Francesc Formiga, Luis Manzano, Harriette G.C. Van Spall, Joan Carles Trullàs

**Affiliations:** ^1^ Division of Cardiology, Department of Medicine UT Southwestern Medical Center Dallas TX USA; ^2^ Division Cardiology Texas Heart Institute Houston TX USA; ^3^ Baylor Scott and White Research Institute Dallas TX USA; ^4^ The Heart Hospital, Baylor Scott and White Plano TX USA; ^5^ Internal Medicine Department Hospital de Manises, Avinguda de la Generalitat, Valenciana Valencia Spain; ^6^ Internal Medicine Department Hospital Universitario de Getafe, Carretera de Madrid‐Toledo Madrid Spain; ^7^ Heart Failure Unit, Internal Medicine Department Hospital Universitari Arnau de Villanova, Institut de Recerca Biomédica (IRBLleida) Lleida Spain; ^8^ Internal Medicine Department Hospital Universitari de Bellvitge, IDIBELL, L'Hospitalet de Llobregat Barcelona Spain; ^9^ Internal Medicine Department Hospital Universitario Ramón y Cajal, IRYCIS, Universidad de Alcalá Madrid Spain; ^10^ Department of Medicine and Department of Health Research Methods, Evidence, and Impact, Faculty of Health Sciences McMaster University Hamilton ON Canada; ^11^ Population Health Research Institute Hamilton Canada; ^12^ Internal Medicine Department Hospital d'Olot i Comarcal de la Garrotxa Girona Spain; ^13^ Laboratori de Reparació i Regeneració Tissular (TR2Lab), Facultat de Medicina Universitat de Vic ‐ Universitat Central de Catalunya Barcelona Spain

**Keywords:** Acute heart failure, Diuretic resistance, Thiazide, Decongestion

## Abstract

**Aims:**

Diuretic resistance (DR) in acute decompensated heart failure (ADHF) is associated with poor outcomes. The CLOROTIC trial demonstrated hydrochlorothiazide (HCTZ) with intravenous furosemide improved weight loss in ADHF. We assessed performance of the BAN‐ADHF DR score and association between DR risk and HCTZ treatment response.

**Methods and results:**

We included participants from CLOROTIC and assessed BAN‐ADHF performance in identifying lowest net diuretic efficiency phenogroup, as identified with a random forest classifier. Participants were stratified by the BAN‐ADHF score (≤12: low DR risk and >12: high DR risk) to compare treatment effect of HCTZ versus placebo across DR risk. Outcomes were weight change and area under the curve (AUC) of dyspnoea visual analogue scale (VAS) at 72/96 h, fluid loss at 72 h, and 30/90‐day mortality. Among 220 participants (50.9% male, mean age 83 years), the BAN‐ADHF score demonstrated good performance in identifying low net diuretic efficiency phenogroup (area under the receiver operating characteristic curve: 0.81). Weight change and VAS AUC at 72 h was similar across DR risk strata, however, participants with high versus low DR risk had lower fluid loss at 72 h (686 vs. 889 ml, *p* = 0.003), higher 30‐day (11.1% vs. 2.4%, *p* = 0.035) and 90‐day mortality (23.0% vs. 10.6%, *p* = 0.033). The treatment effect of HCTZ (vs. placebo) on weight change was similar across DR risk (*p*_int_ = 0.75 at 72 h; *p*_int_ = 0.50 at 96 h). DR risk modified efficacy of HCTZ versus placebo on dyspnoea VAS, with high (vs. low) DR risk having attenuated improvement at 72 h (−236 vs. 476 mm; *p*_int_ = 0.03) and 96 h (−400 vs. 777 mm; *p*_int_ = 0.001).

**Conclusions:**

The BAN‐ADHF score demonstrated good performance in identifying DR, and high DR risk was associated with worse outcomes. HCTZ treatment response on weight loss was similar across DR risk, but improvement in dyspnoea with HCTZ was attenuated in high DR risk.

## Introduction

Acute decompensated heart failure (ADHF) represents a growing public health challenge, accounting for over one million hospitalizations annually in the United States.^1^ Outcomes following hospitalization for ADHF are poor, with 30‐day readmission rates exceeding 20% with significant short‐ and long‐term mortality risk.^2^ A major challenge in the management of ADHF is diuretic resistance (DR), characterized by inadequate natriuresis and volume removal despite appropriate loop diuretic therapy.^3^, ^4^ DR is highly prevalent among patients hospitalized with ADHF and is associated with prolonged hospital stays, incomplete decongestion, and increased mortality.^5^, ^6^, ^7^

While loop diuretics block sodium reabsorption at the loop of Henle, chronic use may lead to compensatory sodium retention in the distal tubule.^8^, ^9^, ^10^, ^11^ Thus, sequential nephron blockade with combination diuretic therapy represents a potential strategy to overcome DR.^12^ Recently, the CLOROTIC trial demonstrated that the addition of the thiazide diuretic hydrochlorothiazide (HCTZ) to intravenous (IV) furosemide improved weight loss among hospitalized patients with ADHF who received high doses of outpatient oral furosemide.^13^ However, while high outpatient diuretic requirements may suggest DR, the trial did not specifically target patients with DR.

Identifying patients most likely to benefit from combination diuretic therapy remains challenging. The recently validated BAN‐ADHF diuretic efficiency score uses readily available clinical parameters at admission to identify patients at high risk for DR.^14^ However, whether DR risk, as determined by the BAN‐ADHF score, modifies the efficacy of combination diuretic therapy among patients with ADHF remains unknown. To address this knowledge gap, we conducted a post‐hoc analysis of the CLOROTIC trial to validate the performance of the BAN‐ADHF score in identifying patients with DR and to evaluate whether baseline DR, assessed by the BAN‐ADHF score, modified the treatment of HCTZ when added to IV loop diuretic therapy.

## Methods

### Study design and population

This post‐hoc analysis included participants from the CLOROTIC trial, a multicentre, randomized, double‐blind, placebo‐controlled study which evaluated the efficacy of adding HCTZ versus placebo to IV furosemide in ADHF. Details of the CLOROTIC trial have been previously described.^13^, ^15^ Briefly, eligible patients were adults hospitalized for ADHF who were receiving chronic oral loop diuretic therapy (≥80 mg furosemide or equivalent) for at least 1 month prior to admission. Key exclusion criteria included clinical instability on admission (acute coronary syndrome, cardiogenic shock, or requiring intensive care), recent inotrope use, renal replacement therapy, thiazide diuretic treatment within the previous month, potassium ≤2.5 mmol/L or sodium ≤125 mmol/L at randomization.

Participants were randomized 1:1 within 24 h of admission to receive either oral HCTZ or placebo for 5 days in addition to IV furosemide. HCTZ dosing was adjusted based on estimated glomerular filtration rate (eGFR) categories: >50 ml/min/1.73 m^2^ (25 mg daily), 20–50 ml/min/1.73 m^2^ (50 mg daily), and <20 ml/min/1.73 m^2^ (100 mg daily). Standardized IV furosemide dosing was implemented across all participating centres according to a pre‐specified algorithm. Participants were monitored during the intervention period and followed for 90 days after discharge.

### BAN‐ADHF risk score and net diuretic efficiency phenomapping approach

We employed a previously validated random forest classifier to identify net diuretic efficiency phenogroups based on urine output in ml per mg of IV furosemide.^14^ We classified participants into three distinct phenogroups based on their net diuretic efficiency profiles, with phenogroup 1 representing lowest net diuretic efficiency. We then evaluated the performance of the integer BAN‐ADHF score in identifying the lowest net diuretic efficiency phenogroup (phenogroup 1). The BAN‐ADHF score is a validated clinical risk tool developed using machine learning approaches to assess the risk of low net diuretic efficiency among patients with ADHF. The score, which ranges from 0 to 26, is calculated using clinical parameters from the index admission. Covariates include blood urea nitrogen, creatinine, natriuretic peptide levels, atrial fibrillation, diastolic blood pressure, hypertension, home diuretic dose, and heart failure hospitalization history (online supplementary *Table* *S1*).

### Outcomes

All clinical outcomes were prospectively collected and adjudicated as part of the CLOROTIC trial protocol. The primary efficacy endpoints were changes in body weight and patient‐reported dyspnoea (assessed as area under the curve [AUC] of the visual analogue scale [VAS]) from baseline to 72 h after randomization. The VAS score ranged from 0 to 100, with higher scores indicating less dyspnoea. Secondary endpoints included weight change and dyspnoea VAS at 96 h, weight loss per 40 mg furosemide at both 72 and 96 h, net fluid loss at 72 h, cumulative IV furosemide dose administered from enrolment through 72 h, and net diuretic efficiency (defined as net urine output divided by cumulative IV furosemide dose [ml/mg]) at 72 h. Clinical outcomes included length of hospital stay and 30‐ and 90‐day all‐cause mortality and rehospitalization. Safety endpoints included changes in renal function (defined as serum creatinine increase >26.5 μmol/L or eGFR decrease ≥50% from admission) and electrolyte disturbances (hypokalaemia defined as potassium ≤3.0 mmol/L and hyponatraemia as sodium ≤130 mmol/L).

### Statistical analysis

Summary statistics were reported as median (interquartile range [IQR]) for continuous variables and frequencies (percentages) for categorical variables. Baseline characteristics were compared across BAN‐ADHF risk strata using Kruskal–Wallis tests for continuous variables and chi‐square or Fisher's exact tests for categorical variables. The association between BAN‐ADHF score and net diuretic efficiency was assessed using linear mixed‐effects models with random patient intercepts. We identified the optimal BAN‐ADHF threshold for predicting net diuretic efficiency at 72 h using receiver operating characteristic (ROC) curve analysis and Youden's index maximization, which established a score >12 as the best cutoff value for identifying DR.^16^ Models were evaluated with the BAN‐ADHF score both as a continuous variable (per 1‐unit increase) and categorical variable (>12 vs. ≤12). The performance of the BAN‐ADHF score in identifying the lowest net diuretic efficiency phenogroup was evaluated using the area under the receiver operating characteristic curve (AUROC). We also assessed the performance of the BAN‐ADHF score for predicting participants in the lowest quartile of net diuretic efficiency (as defined above) at 72 h post‐randomization.

The association between HCTZ (vs. placebo) treatment across BAN‐ADHF risk strata and outcomes was analysed using linear mixed‐effects models for continuous outcomes (weight change, VAS, net diuretic efficiency) adjusted for baseline measurements, Cox proportional hazards models for time‐to‐event outcomes (mortality, rehospitalization) adjusted for baseline weight, and logistic regression models for safety outcomes adjusted for baseline laboratory value. Interaction terms between treatment assignment (HCTZ vs. placebo) and BAN‐ADHF risk groups were tested to assess differential treatment effects. The significance of interactions was evaluated by comparing nested models with and without interaction terms using likelihood ratio tests. Analyses were performed using R version 4.1.3, with two‐sided *p*‐values <0.05 considered statistically significant.

## Results

Among 230 participants enrolled in the CLOROTIC trial, 220 (96%) had complete data available for BAN‐ADHF score calculation and were included in this analysis. The median BAN‐ADHF score was 14 (interquartile range: 11–16) (online supplementary *Figure* *S1*), with 135 (61.4%) participants classified as high DR risk (BAN‐ADHF >12). Baseline characteristics stratified by DR risk are shown in *Table* *1*. Participants with high DR risk were more likely to be male (62.2% vs. 32.9%) and have heart failure with reduced ejection fraction (34.1% vs. 20.0%). Those with higher DR risk score had a higher prevalence of diabetes (63.7% vs 42.4%) and ischaemic heart disease (40.0% vs. 17.9%). As expected based on the BAN‐ADHF scoring algorithm, participants with high (vs. low) DR risk had worse renal function, higher natriuretic peptide levels, higher doses of diuretics at home, and greater prevalence of atrial fibrillation and hypertension. There were no significant differences in age, New York Heart Association class III–IV symptoms, or body mass index between risk groups.

**Table 1:** Baseline characteristics stratified by diuretic resistance risk

Characteristic	Low DR risk (*n* = 85)	High DR risk (*n* = 135)	*p*‐value
Age, years	84 [78–87]	83 [76–87]	0.310
Male sex	28 (32.9)	84 (62.2)	<0.001
White race	85 (100.0)	134 (99.3)	1.000
NYHA class			0.536
I	1 (1.2)	5 (3.7)	
II	34 (40.0)	45 (33.3)	
III	41 (48.2)	72 (53.3)	
IV	9 (10.6)	13 (9.6)	
LVEF			0.030
≥50%	64 (75.3)	78 (57.8)	
41–49%	4 (4.7)	11 (8.1)	
≤40%	17 (20.0)	46 (34.1)	
Ischaemic cardiomyopathy	15 (17.9)	54 (40.0)	0.001
Diabetes	36 (42.4)	86 (63.7)	0.003
Anaemia	30 (35.3)	69 (51.1)	0.031
Stroke	10 (11.8)	19 (14.1)	0.773
COPD	17 (20.0)	33 (24.4)	0.548
Atrial fibrillation	50 (58.8)	102 (75.6)	0.014
Hypertension	70 (82.4)	126 (93.3)	0.020
Prior HF hospitalization	36 (42.4)	95 (70.4)	<0.001
Creatinine, mg/dl	1.09 [0.86–1.27]	1.60 [1.40–1.89]	<0.001
Blood urea nitrogen, mg/dl	58.00 [43.00–72.00]	95.00 [72.00–115.50]	<0.001
eGFR, ml/min/1.73 m^2^	57.00 [44.00–71.00]	38.00 [29.90–47.00]	<0.001
NT‐proBNP, pg/ml	2285 [1239–4338]	5913 [2147–10 063]	<0.001
Potassium, mmol/L	4.20 [3.87–4.70]	4.32 [3.90–4.72]	0.195
Home diuretic dose, mg	80 [80–80]	90 [80–120]	<0.001
Beta‐blocker	45 (52.9)	87 (64.4)	0.120
ACE inhibitor/ARB	51 (60.0)	71 (52.6)	0.349
HCTZ dose			<0.001
25 mg	43 (51.2)	23 (17.0)	
50 mg	40 (47.6)	104 (77.0)	
100 mg	1 (1.2)	8 (5.9)	
BAN‐ADHF score^a^	10.0 [9.0–11.0]	15.0 [14.0–17.0]	<0.001

Values presented as *n* (%) for categorical variables and median [interquartile range] for continuous variables.

ACE, angiotensin‐converting enzyme; ARB, angiotensin receptor blocker; COPD, chronic obstructive pulmonary disease; DR, diuretic resistance; eGFR, estimated glomerular filtration rate; HCTZ, hydrochlorothiazide; HF, heart failure; LVEF, left ventricular ejection fraction; NT‐proBNP, N‐terminal pro‐B‐type natriuretic peptide; NYHA, New York Heart Association.

^a^ BAN‐ADHF score components include blood urea nitrogen, NT‐proBNP, atrial fibrillation, diastolic blood pressure, hypertension, home diuretic dose, and prior HF hospitalization within 12 months. Low DR risk defined as BAN‐ADHF score ≤12; high DR risk defined as BAN‐ADHF score >12.

### Validation of the BAN‐ADHF score

**Figure 1:**
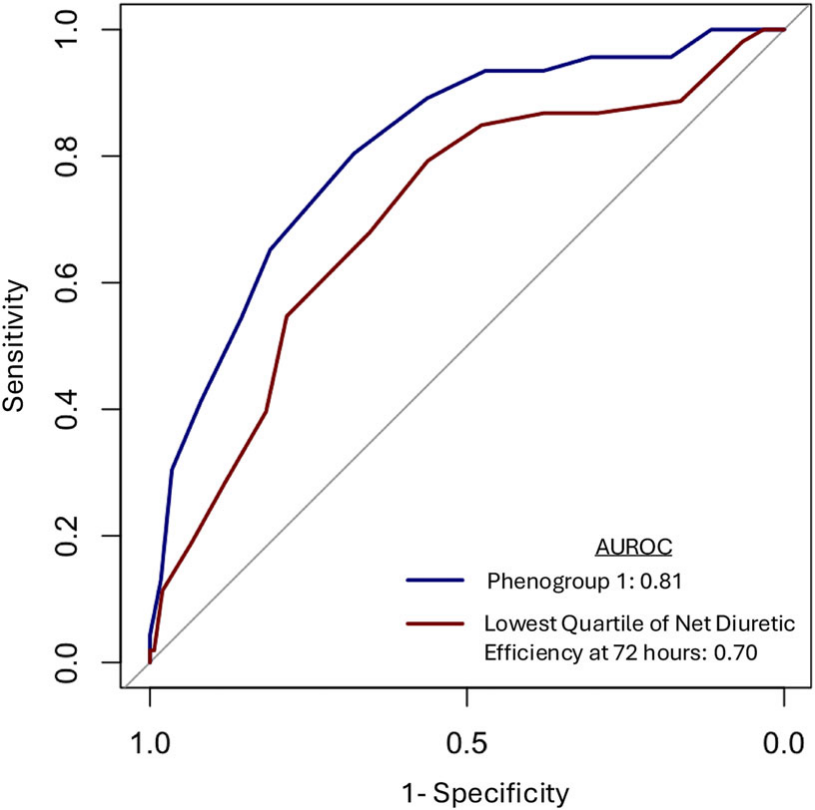
Performance of the BAN‐ADHF score in assessing low net diuretic efficiency phenogroup and lowest quartile of net diuretic efficiency at 72 h. Receiver operating characteristic curves demonstrating the discriminative ability of the BAN‐ADHF score for identifying the lowest net diuretic efficiency phenogroup (phenogroup 1) and lowest quartile of net diuretic efficiency at 72 h. AUROC, area under the receiver operating characteristic curve.

We identified three distinct phenogroups based on net diuretic efficiency profiles. Phenogroup 1 represented patients with the lowest net diuretic efficiency (online supplementary *Figure*  *S2*). The BAN‐ADHF score demonstrated an AUROC of 0.81 in identifying the lowest net diuretic efficiency phenogroup (*Figure* *1*). Additionally, we evaluated the performance of the BAN‐ADHF score in identifying participants with DR at 72 h, and the score demonstrated good discriminatory ability (AUROC: 0.70). There was a significant inverse association between the BAN‐ADHF score and net diuretic efficiency when assessed both continuously (β [95% confidence interval, CI]: −0.18 ml/mg [−0.28, −0.08] per 1‐unit increase in BAN‐ADHF score, *p* < 0.001) and categorically (β [95% CI]: −1.29 [−2.04, −0.53] ml/mg for BAN‐ADHF score >12, *p* = 0.001) (online supplementary *Table* *S2*).

### Outcomes stratified by BAN‐ADHF risk strata

There were no significant differences in the co‐primary outcomes of weight change at 72 h between high and low DR risk groups (−1.70 kg [IQR: −3.65, −0.50] vs. −2.00 kg [IQR: −3.90 to −0.60]; *p* = 0.31) or at 96 h (−2.00 kg [IQR: −4.00 to −0.70] vs. −2.00 kg [IQR: −4.50 to −0.90]; *p* = 0.62) (*Table* *2*). Similarly, patient‐reported dyspnoea assessed by AUC of the VAS showed no significant differences between high and low DR risk groups at 72 h (900 mm [IQR: 330−1530] vs. 900 mm [IQR: 480−1620]; *p* = 0.35) or at 96 h (1380 mm [IQR: 480−2460] vs. 1440 mm [IQR: 900−2760]; *p* = 0.21). Participants with high DR risk demonstrated significantly lower net fluid loss at 72 h (686 ml [IQR: 430−973] vs. 889 ml [IQR: 590−1158]; *p* = 0.003), required higher cumulative furosemide doses (400 mg [IQR: 300−520] vs. 320 mg [IQR: 275−400]; *p* = 0.001), and had lower net diuretic efficiency (1.85 ml/mg [IQR: 1.11−3.15] vs. 2.50 ml/mg [IQR: 1.78−4.13]; *p* < 0.001) compared to those with low DR risk.

**Table 2:** Outcomes for hydrochlorothiazide versus placebo by diuretic resistance risk

Outcome	Low DR risk (*n* = 85)	High DR risk (*n* = 135)	*p‐*value
Primary outcomes			
Weight change at 72 h (kg)	−2.00 [−3.90 to −0.60]	−1.70 [−3.65 to −0.50]	0.310
AUC for dyspnoea VAS at 72 h (mm)	900 [480–1620]	900 [330–1530]	0.352
Secondary outcomes			
Weight change at 96 h (kg)	−2.00 [−4.50 to −0.90]	−2.00 [−4.00 to −0.70]	0.618
AUC for dyspnoea VAS 96 h (mm)	1440 [900–2760]	1380 [480–2460]	0.205
Weight loss (kg) per 40 mg furosemide at 72 h	−0.36 [−0.67 to −0.11]	−0.25 [−0.56 to −0.05]	0.116
Weight loss (kg) per 40 mg furosemide at 96 h	−0.27 [−0.67 to −0.14]	−0.23 [−0.56 to −0.05]	0.195
Net fluid loss at 72 h (ml)	888.89 [590.00–1158.33]	685.71 [430.30–973.21]	0.003
Cumulative furosemide dose at 72 h (mg)	320 [275–400]	400 [300–520]	0.001
Net diuretic efficiency at 72 h (ml/mg)	2.50 [1.78–4.13]	1.85 [1.11–3.15]	<0.001
Clinical outcomes			
Length of stay (days)	7.0 [5.0–10.0]	7.0 [6.0–11.0]	0.253
All‐cause mortality at 30 days	2 (2.4)	15 (11.1)	0.035
All‐cause mortality at 90 days	9 (10.6)	31 (23.0)	0.033
All‐cause readmission at 30 days	14 (16.5)	29 (21.5)	0.461
All‐cause readmission at 90 days	27 (31.8)	51 (37.8)	0.445
Safety outcomes			
Increase in creatinine >0.3 mg/dl	22 (25.9)	47 (34.8)	0.215
Decrease in eGFR >50%	2 (2.4)	0 (0.0)	0.289
Sodium <130 mmol/L	5 (5.9)	11 (8.1)	0.716
Potassium <3.0 mmol/L	4 (4.7)	11 (8.1)	0.477

Values presented as median [interquartile range] for continuous variables and *n* (%) for categorical variables.

Low DR risk is defined as BAN‐ADHF score ≤12; high DR risk is defined as BAN‐ADHF score >12. Higher VAS scores indicated less dyspnoea. Net fluid loss is calculated as the difference between total fluid intake and output. Net diuretic efficiency is defined as net urine output (ml) per mg of intravenous furosemide administered.

AUC, area under the curve; DR, diuretic resistance; eGFR, estimated glomerular filtration rate; VAS, visual analogue scale.

Among clinical outcomes, the median length of stay was similar between groups (7.0 days [IQR: 6.0−11.0] vs. 7.0 days [IQR: 5.0−10.0]; *p* = 0.25). However, the high DR risk group demonstrated significantly higher 30‐day (11.1% vs. 2.4%, *p* = 0.04) and 90‐day mortality (23.0% vs. 10.6%, *p* = 0.03). Rehospitalization rates were numerically higher in the high DR risk group at both 30 days (21.5% vs. 16.5%, *p* = 0.46) and 90 days (37.8% vs. 31.8%, *p* = 0.45). Among safety outcomes, there were no significant differences in impaired renal function, severe hyponatraemia, or hypokalaemia across DR risk groups.

### Treatment effect modification by diuretic resistance risk

The effect of HCTZ versus placebo on weight loss was not modified by DR risk (*p*_interaction_ treatment*DR risk = 0.75 at 72 h and *p*_interaction_ treatment*DR risk = 0.50 at 96 h). The median difference in weight change between HCTZ and placebo arms was similar between DR risk groups at both 72 h (median difference [95% CI] for low vs. high DR risk: −4.8 kg [−13.0 to 3.2], *p* = 0.24 vs. −3.0 kg [−8.9 to 3.0], *p* = 0.33) and 96 h (median difference [95% CI] for low vs. high DR risk: −5.4 kg [−14.0 to 2.6], *p* = 0.18 vs. −3.3 kg [−9.3 to 2.7], *p* = 0.28) (*Table* *3*). In contrast, DR risk significantly modified the treatment efficacy of HCTZ versus placebo on patient‐reported dyspnoea (*Table* *3*). At 72 h, participants with low DR risk demonstrated a trend towards improvement in the AUC of the dyspnoea VAS with HCTZ versus placebo (median difference [95% CI]: 476 mm [−34 to 987], *p* = 0.07), while those with high DR risk experienced trend towards worsening dyspnoea with HCTZ versus placebo (median difference [95% CI]: −236 mm [−669 to 197], *p* = 0.28; *p*_interaction_ treatment*DR risk = 0.03). This differential response was even more pronounced for the treatment effect of HCTZ versus placebo on the AUC of the dyspnoea VAS at 96 h (median difference [95% CI] for low vs. high DR risk: 777 mm [266−1287], *p* = 0.003 vs. −400 mm [−833 to 33], *p* = 0.07; *p*_interaction_ treatment*DR risk = 0.001). The effect of HCTZ versus placebo on net diuretic efficiency at 72 h was not modified by DR risk (*p*_interaction_ treatment*DR risk = 0.84). HCTZ improved net diuretic efficiency in patients with high DR risk (mean difference: 0.55 ml/mg, 95% CI 0.03−1.10, *p* = 0.04) with a similar magnitude but non‐significant effect in those with low DR risk (mean difference: 0.69 ml/mg, 95% CI −0.36 to 1.7, *p* = 0.20). There was no significant effect modification by DR risk between HCTZ versus placebo on adverse clinical outcomes and safety outcomes (*Table* *4*).

**Table 3:** Treatment effects of hydrochlorothiazide versus placebo on primary outcomes and net diuretic efficiency by diuretic resistance risk groups

	Placebo	HCTZ	Median difference	*p*‐value	*p*_int_
Dyspnoea VAS AUC at 72 h (mm)^a^					
Low DR risk	919 (592–1247)	1395 (1004–1787)	476 (−34 to 987)	0.07	0.03
High DR risk	1027 (706–1348)	791 (499–1082)	−236 (−669 to 197)	0.28	
Dyspnoea VAS AUC at 96 h (mm)^a^					
Low DR risk	1472 (1145–1800)	2249 (1858–2641)	777 (266–1287)	0.003	0.001
High DR risk	1633 (1313–1954)	1233 (942–1524)	−400 (−833 to 33)	0.07	
Weight at 72 h (kg)					
Low DR risk	77 (72–82)	72 (66–78)	−4.8 (−13 to 3.2)	0.24	0.75
High DR risk	80 (75–84)	77 (73–81)	−3.0 (−8.9 to 3)	0.33	
Weight at 96 h (kg)					
Low DR risk	77 (72–82)	72 (65–78)	−5.4 (−14 to 2.6)	0.18	0.50
High DR risk	80 (75–84)	76 (72–80)	−3.3 (−9.3 to 2.7)	0.28	
Net diuretic efficiency at 72 h (ml/mg)^b^					
Low DR risk	3.3 (2.6–4)	4 (3.2–4.8)	0.69 (−0.36 to 1.70)	0.20	0.84
High DR risk	2.1 (1.7–2.4)	2.6 (2.3–3)	0.55 (0.03–1.10)	0.04	

AUC, area under the curve; DR, diuretic resistance; HCTZ, hydrochlorothiazide; VAS, visual analogue scale.

Values shown are estimated marginal means and 95% confidence intervals using linear mixed‐effects models with random patient intercepts, adjusted for baseline values of the respective outcomes. The median difference represents the difference between HCTZ and placebo groups. *P*_interaction_ assessed the interaction of treatment by DR risk.

High DR risk was determined by a BAN‐ADHF score >12, while low DR risk was determined by a BAN‐ADHF score ≤12.

^a^ Measured as the mean AUC of daily VAS assessments by treatment group from randomization through 72 and 96 h, where increasing values represent improvements in dyspnoea.

^b^ Net diuretic efficiency at 72 h defined as cumulative net urine output (ml) divided by cumulative furosemide dose (mg) administered over 72 h.

**Table 4:** Treatment effect of hydrochlorothiazide versus placebo on clinical and safety outcomes by diuretic resistance risk

Outcome	Low DR risk			*p*‐value	High DR risk			*p*‐value	*p*_int_
	Placebo (*n* = 50)	HCTZ (*n* = 35)	HR/OR (95% CI)		Placebo (*n* = 61)	HCTZ (*n* = 74)	HR/OR (95% CI)		
All‐cause mortality at 30 days	1 (2.0)	1 (2.9)	HR 1.54 (0.09–25.48)	0.762	6 (9.8)	9 (12.2)	HR 1.43 (0.50–4.08)	0.501	0.945
All‐cause mortality at 90 days	5 (10.0)	4 (11.4)	HR 1.21 (0.32–4.56)	0.481	14 (23.0)	17 (23.0)	HR 1.10 (0.54–2.25)	0.792	0.978
All‐cause readmission at 30 days	8 (16.0)	6 (17.1)	HR 1.05 (0.36–3.04)	0.780	9 (14.8)	20 (27.0)	HR 2.08 (0.95–4.57)	0.068	0.301
All‐cause readmission at 90 days	15 (30.0)	12 (34.3)	HR 1.17 (0.54–2.53)	0.691	22 (36.1)	29 (39.2)	HR 1.27 (0.73–2.21)	0.398	0.850
Impaired renal function^a^	7 (14.0)	15 (42.9)	OR 4.56 (1.60–12.94)	0.004	11 (18.0)	36 (48.6)	OR 4.31 (1.94–9.61)	<0.001	0.917
Decrease in eGFR >50%	1 (2.0)	1 (2.9)	OR 1.73 (0.10–30.37)	0.708	0 (0.0)	0 (0.0)	OR 1.00 (0–∞)	1.0	1.0
Sodium <130 mmol/L	2 (4.0)	3 (8.6)	OR 2.32 (0.34–15.90)	0.390	4 (6.6)	7 (9.5)	OR 1.97 (0.36–10.83)	0.437	0.618
Potassium <3.0 mmol/L	1 (2.0)	3 (8.6)	OR 4.45 (0.40–50.04)	0.227	2 (3.3)	9 (12.2)	OR 4.07 (0.79–21.09)	0.094	0.949

Data shown are *n* (%).

CI, confidence interval; DR, diuretic resistance; eGFR, estimated glomerular filtration rate; HCTZ, hydrochlorothiazide; HR, hazard ratio; OR, odds ratio.

OR/HR compare HCTZ versus placebo. Clinical outcomes are presented as HR derived from Cox proportional hazards models adjusted for baseline weight. Safety outcomes are presented as OR from logistic regression models adjusted for the corresponding baseline laboratory values (creatinine, eGFR, sodium, or potassium). High DR risk was determined by a BAN‐ADHF score >12, while low DR risk was determined by a BAN‐ADHF score ≤12. *P*_interaction_ assessed the interaction of treatment by DR risk.

^a^ Defined as increase in creatinine >26.5 μmol/L from baseline.

## Discussion

In this post‐hoc analysis of the CLOROTIC trial, we demonstrated that the BAN‐ADHF score effectively identified patients at high risk for DR, with good discrimination for identification of both the lowest net diuretic efficiency phenogroup and DR at 72 h. Participants classified as high DR risk by the BAN‐ADHF score demonstrated significantly lower net fluid loss, higher cumulative IV furosemide doses, and lower net diuretic efficiency compared to those with low DR risk. Additionally, patients with high (vs. low) risk for DR experienced significantly higher mortality at both 30 days and 90 days. Treatment response to HCTZ on weight did not change across DR risk group, however, DR risk significantly modified the treatment effect of HCTZ on dyspnoea improvement, with participants with high DR risk having attenuated improvement in dyspnoea despite similar reductions in weight compared to low DR risk patients (*Graphical Abstract*). This dissociation between objective and subjective measures of decongestion suggests that the pathophysiology of DR may impact symptom relief through mechanisms beyond achieved diuresis.

The BAN‐ADHF score was originally developed using machine learning approaches to identify patients at high risk for DR, demonstrating that patients with high DR risk had substantially lower urine output despite equivalent diuretic doses and experienced worse clinical outcomes.^14^ Initial external validation in a real‐world cohort at a large academic medical centre confirmed the score's ability to assess both DR risk and its association with adverse clinical outcomes.^17^ Our analysis extends this prior work by externally validating the BAN‐ADHF score in a well phenotyped randomized controlled trial population of patients with ADHF. The BAN‐ADHF score demonstrated good discrimination for identifying the lowest net diuretic efficiency phenogroup and DR at 72 h. This robust performance is particularly noteworthy given both the standardized diuretic protocol administered to participants and the CLOROTIC population's higher baseline risk profile, comprised exclusively of patients with chronic heart failure requiring substantial maintenance diuretic therapy (≥80 mg oral furosemide daily) and a high burden of baseline chronic kidney disease. The physiologic hallmarks of DR were evident in high‐risk patients, who demonstrated significantly lower net fluid loss despite receiving a higher cumulative dose of protocol‐directed IV furosemide, resulting in reduced net diuretic efficiency. This translated into worse clinical outcomes, with high (vs. low) DR risk patients experiencing substantially greater rates of 30‐day and 90‐day mortality. While length of stay was not different across groups, this likely reflects the trial protocol detailing at least 5 days of inpatient diuretic therapy in both groups. These findings provide robust validation of the BAN‐ADHF score's ability to identify a distinct phenotype of DR associated with markedly worse prognosis, even in a clinical trial population receiving standardized diuretic therapy.

Sequential nephron blockade with thiazides targets distinct sodium transport mechanisms in the distal convoluted tubule, potentially overcoming the compensatory sodium retention observed with loop diuretic treatment that characterizes DR.^18^ This mechanistic rationale may explain why treatment with HCTZ achieved similar improvements in weight loss and net diuretic efficiency regardless of DR risk. Notably, patients with high DR risk predominantly received higher HCTZ doses (83% receiving ≥50 mg) compared to those with low DR risk (49% receiving ≥50 mg), which may have aided in volume removal in those with high DR risk. However, patients with high (vs. low) DR risk demonstrated attenuated improvement in dyspnoea. This dissociation between objective and subjective measures of decongestion is corroborated by prior research that has demonstrated changes in volume status often correlate poorly with improvements in dyspnoea in ADHF.^19^ Our findings indicate that this disconnect may be particularly pronounced in patients with DR. This divergence suggests that the pathophysiology underlying symptom perception in DR extends beyond simple fluid dynamics. The complex neurohormonal and haemodynamic derangements characteristic of advanced DR, including heightened sympathetic activation, reduced cardiac reserve, and impaired kidney function, may influence how patient symptoms respond to decongestive therapy.^20^ These findings challenge the conventional assumption that successful volume removal necessarily translates to symptomatic improvement, particularly in patients with established DR.

Our analysis reveals important insights about identifying patients who are most likely to benefit from combination HCTZ and loop diuretic therapy. Prior analyses of the CLOROTIC trial demonstrated a clinically meaningful gradient in treatment response across eGFR distribution, with greater diuretic response among patients with higher eGFR, particularly at levels >60 ml/min/1.73 m^2^, with a significant treatment interaction noted between continuous eGFR and treatment intervention for the outcome of weight change by 72 h (*p*_interaction_ = 0.002) and 96 h (*p*_interaction_ = 0.016).^21^ However, eGFR is only one of the biological factors that influences diuretic response and may be insufficient for identifying optimal candidates for combination diuretic therapy. The BAN‐ADHF score, which incorporates multiple dimensions of cardio‐renal dysfunction beyond eGFR alone, may provide more refined risk stratification to identify patients most likely to achieve meaningful clinical benefit from HCTZ therapy, particularly by capturing additional factors that influence diuretic responsiveness beyond kidney function alone. Moreover, the higher rates of worsening renal function we observed with HCTZ treatment in those with high DR risk, though not statistically significant, raise important safety concerns that warrant further study, especially considering the increased risk of elevated creatinine observed with HCTZ therapy seen in the overall CLOROTIC trial.^13^

### Clinical implications

The BAN‐ADHF score represents a readily implementable tool for improving ADHF management, requiring only routine clinical parameters to calculate an integer‐based risk score. Its simplicity makes it particularly suitable for integration into existing care pathways and electronic health record systems, allowing for rapid risk stratification and treatment planning. Healthcare systems could incorporate the score into admission order sets and clinical decision support tools to guide initial therapy selection and monitoring strategies and inform providers of the heightened risk of post‐discharge adverse outcomes among those with high DR risk.

Identification of DR risk may have direct therapeutic implications on ADHF management. Our findings indicate that incorporating DR risk assessment could shift treatment selection in the expanding therapeutic landscape of ADHF. While standard approaches often rely on sequential addition of diuretics based primarily on volume status and renal function, the differential symptomatic response observed with HCTZ across DR risk strata suggests the need for more sophisticated treatment algorithms. Given our observation that patients with high DR risk show attenuated symptomatic improvement with HCTZ despite adequate volume removal, clinicians may consider alternative first‐line combination strategies that target the complex pathophysiology of DR. Extending this concept to the post‐discharge setting, our group has previously demonstrated that the BAN‐ADHF score can identify patients with DR who achieve significantly higher net diuretic efficiency with subcutaneous furosemide compared to oral furosemide in the vulnerable post‐hospitalization period.^22^ This suggests that DR risk assessment using the BAN‐ADHF score may have utility across the continuum of heart failure care, from guiding inpatient therapies to informing outpatient management strategies after discharge.

The therapeutic armamentarium of ADHF now includes multiple evidence‐based options, such as acetazolamide, natriuresis‐guided loop diuretic therapy, and sodium–glucose co‐transporter 2 inhibitors, and each target distinct mechanisms underlying volume overload.^23^, ^24^, ^25^, ^26^ Future clinical trials should prospectively stratify patients by DR risk and evaluate both objective and patient‐reported outcomes to determine which therapeutic strategies provide optimal benefit for specific DR phenotypes. Such evidence would support the development of personalized treatment algorithms that consider not just the presence of DR, but its severity and associated pathophysiological features.

This post‐hoc analysis offers several key strengths, including robust validation of the BAN‐ADHF score in a well‐characterized clinical trial population with extended follow‐up to evaluate clinical outcome differences. However, important limitations must be acknowledged. The small sample size of the CLOROTIC trial limited statistical power. Our findings may not be generalized to patients with newly diagnosed heart failure, who are diuretic naïve, or have low outpatient doses of loop diuretic, as all participants in the CLOROTIC trial had chronic heart failure requiring moderate‐to‐high doses of loop diuretics before admission. Further, there was a high prevalence of heart failure with preserved ejection fraction in the cohort, which may limit generalizability to patients with a reduced ejection fraction. Finally, the post‐hoc calculation of the BAN‐ADHF score limits our ability to determine whether score‐guided therapy would improve clinical outcomes.

## Conclusion

In this analysis of the CLOROTIC trial, we demonstrated that the BAN‐ADHF score effectively identifies patients hospitalized with ADHF at high risk for DR at increased risk for post‐discharge adverse outcomes. While combination therapy of HCTZ with IV furosemide achieved similar improvements in weight loss regardless of DR risk, patients with high (vs. low) DR risk demonstrated attenuated improvement in dyspnoea. Future research should evaluate whether prospective risk stratification using the BAN‐ADHF score can guide optimal selection of decongestive therapies and improve patient outcomes in ADHF.

**Conflict of interest**: A.P. notes research support from the National Institute on Minority Health and Disparities (R01MD017529), the National Institute of Heart, Lung, and Blood Institute (R21HL169708), American Heart Association, Ultromics, Anumana, SC Pharmaceuticals, SQ Innovation, AstraZeneca, and Roche Diagnostics; serves as a consultant for and/or received honoraria outside of the present study as an advisor/consultant for Northwestern University, Tricog Health Inc, Lilly USA, Rivus, Cytokinetics, Roche Diagnostics, Sarfez Therapeutics, Edwards Lifesciences, Merck, Bayer, Anumana, Novo Nordisk, Alleviant, Pfizer, Abbott, iRhythm, Axon Therapies, Kilele Health, Acorai, Ultromics, Novartis, Idorsia Pharma, Medical AI, Baylor Scott and White Research Institute, Tenax Pharma, Boehringer Ingelheim, Tourmaline Bio, Anumana, Encarda, Astra Zeneca, and Science37; also served as consultant for Palomarin Inc. with stocks compensation. M.W.S. reports honoraria from Idorsia Pharmaceuticals, non‐financial support from Merck, is on the advisory board of descendantsDNA, and is the founder of ReCODE Medical. N.K. has served as a consultant for HeartSciences, Tricog Health, Inc, Science37, and Idorsia Pharmaceuticals. H.V.S has served as a consultant for Bayer, 4TEEN4, Medtronic, and Novo Nordisk. All other authors have nothing to disclose.

## Supporting information


**Appendix S1.** Supporting Information.
