# Supplementary material for: Diuretic resistance and the efficacy of hydrochlorothiazide in acute decompensated heart failure: A post‐hoc analysis of the CLOROTIC trial
Source: Eur J Heart Fail. 2025 Aug 26;27(12):3142–50. doi: 10.1002/ejhf.70002 (PMC12803567; doi:10.1002/ejhf.70002)
Supplement: Supplementary file 1 — Appendix S1. Supporting Information. [file EJHF-27-3142-s001.docx]

**Supplemental Figure 1**: Distribution of BAN-ADHF scores across the study population.


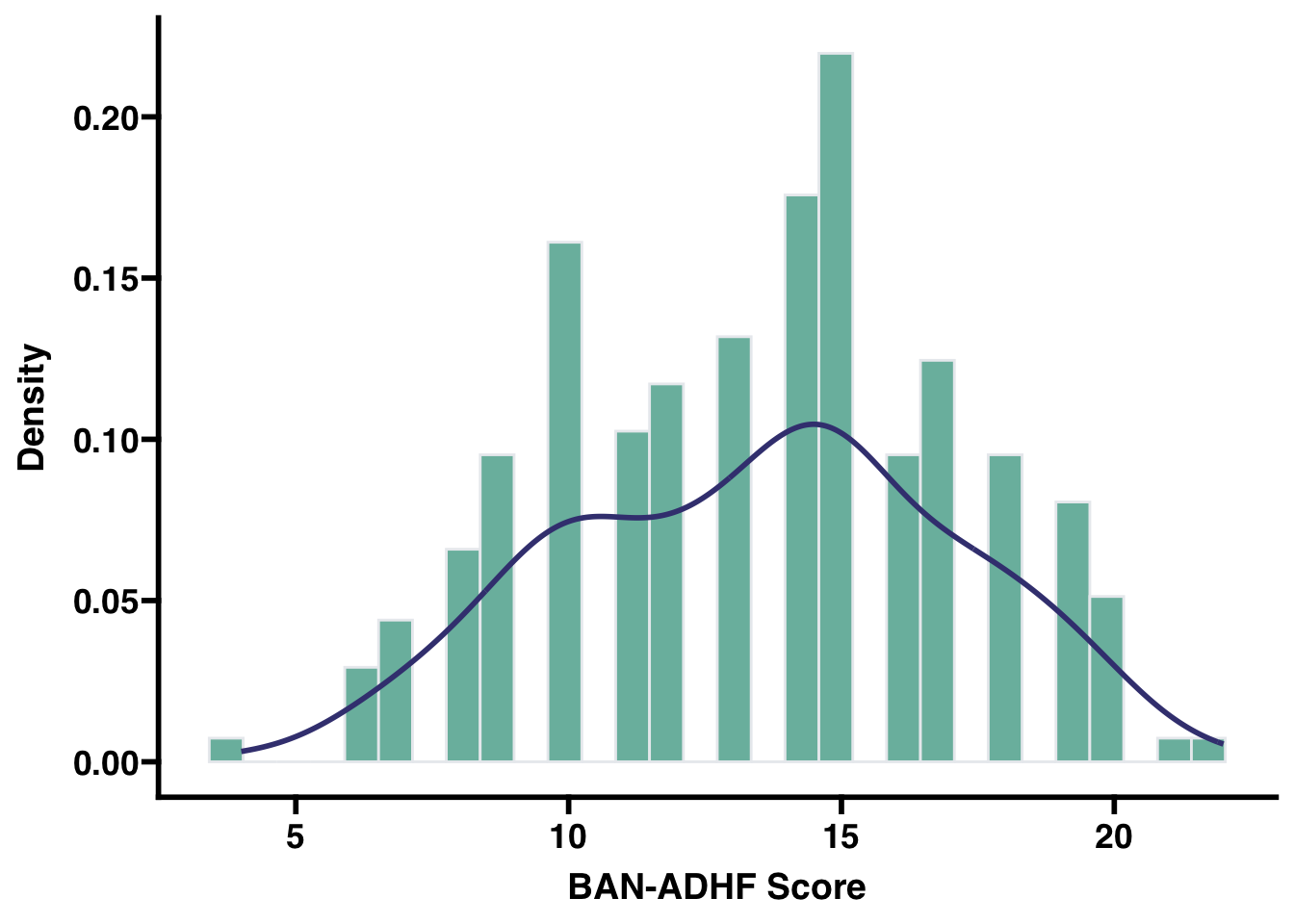


**Supplemental Figure 2**: Net diuretic efficiency phenogroups stratified by treatment arm.


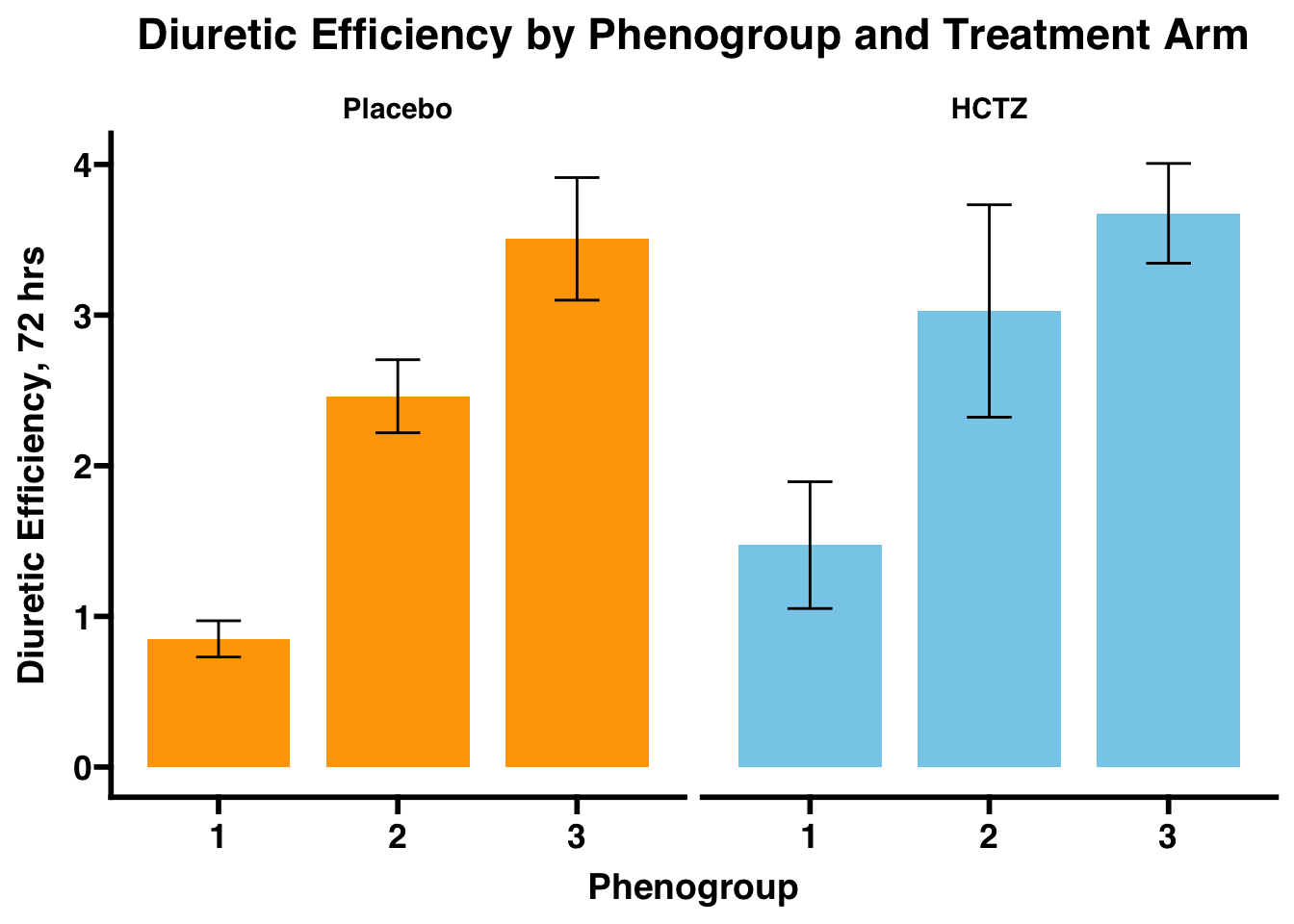


Net diuretic efficiency was defined as cumulative net urine output (mL) per cumulative IV furosemide dose (mg) over 72 hours (mL/mg), with lower values indicating poorer diuretic response. Abbreviations: HTZ - hydrochlorothiazide

**Supplemental Table 1**: Calculation of the BAN-ADHF score to predict the risk of diuretic resistance.

| **Predictor** | **Score** |
| --- | --- |
| Creatinine (mg/dL)  <1.2  1.2-1.59  ≥1.6 | 0  2  4 |
| Diastolic Blood Pressure (mm Hg)  ≥60  50-59  <50 | 0  1  3 |
| Home Diuretic (furosemide equivalent/day)  <120  120-249  ≥250 | 0  3  6 |
| NT-proBNP (pg/mL)  <5000  5000-12000  >12000 | 0  2  4 |
| BUN (mg/dL)  <20  20-39  ≥40 | 0  2  3 |
| Atrial Fibrillation  No  Yes | 0  2 |
| Hypertension  No  Yes | 0  3 |
| Previous HF < 12 months  No  Yes | 0  1 |
| Points are summed across all categories (0-26), with higher scores indicating greater probability of low diuretic efficiency. A BAN-ADHF score >12 indicated diuretic resistance in the present study.  Adapted from Segar et al. *JACC HF.* 2024 Mar;12(3):508-520. doi: 10.1016/j.jchf.2023.09.029  Abbreviations: BUN – blood urea nitrogen, NT-proBNP – N-terminal pro-B-type natriuretic peptide, HF – heart failure | |

**Supplemental Table 2**. Association of BAN-ADHF score with net diuretic efficiency

|  | **Continuous**  **(per 1u increase in BAN-ADHF)** | | **Categorical**  **(BAN-ADHF > 12)** | |
| --- | --- | --- | --- | --- |
|  | **Est (95% CI)** | **P value** | **Est (95% CI)** | **P value** |
| Net diuretic efficiency (mL/mg) at 72 hrs | -0.18  (-0.28, -0.08) | <0.001 | -1.29  (-2.04, -0.53) | 0.001 |
| Lowest quartile of net diuretic efficiency at 72 hrs | 1.24  (1.12, 1.36) | <0.001 | 5.13  (2.27, 11.61) | <0.001 |
| Phenogroup 1 | 1.50  (1.31, 1.73) | <0.001 | 13.52  (4.01, 45.56) | <0.001 |
| Values represent parameter estimates with 95% confidence intervals derived from linear mixed-effects models with random patient intercepts. Models for continuous outcomes were adjusted for their respective baseline values. "Continuous" refers to the parameter estimate per 1-unit increase in BAN-ADHF score, while "Categorical" refers to the parameter estimate comparing high diuretic resistance risk (BAN-ADHF score >12) to low risk (≤12).  Net diuretic efficiency is defined as the cumulative net urine output (mL) per cumulative furosemide dose (mg) measured at 72 hours after randomization (mL/mg), with lower values indicating worse diuretic efficiency. Phenogroup 1 represents patients with the lowest diuretic efficiency profile as determined by a validated random forest classifier (Segar et al., *JACC HF* 2024). | | | | |
